# Developmental control of lateralized neuron size in the nematode *Caenorhabditis elegans*

**DOI:** 10.1186/1749-8104-5-33

**Published:** 2010-12-01

**Authors:** Andrew D Goldsmith, Sumeet Sarin, Shawn Lockery, Oliver Hobert

**Affiliations:** 1 Howard Hughes Medical Institute, Department of Biochemistry and Molecular Biophysics, Columbia University Medical Center, New York, NY 10032, USA; 2 Institute of Neuroscience, University of Oregon, Eugene, OR 97403, USA

## Abstract

**Background:**

Nervous systems are generally bilaterally symmetric on a gross structural and organizational level but are strongly lateralized (left/right asymmetric) on a functional level. It has been previously noted that in vertebrate nervous systems, symmetrically positioned, bilateral groups of neurons in functionally lateralized brain regions differ in the size of their soma. The genetic mechanisms that control these left/right asymmetric soma size differences are unknown. The nematode *Caenorhabditis elegans *offers the opportunity to study this question with single neuron resolution. A pair of chemosensory neurons (ASEL and ASER), which are bilaterally symmetric on several levels (projections, synaptic connectivity, gene expression patterns), are functionally lateralized in that they express distinct chemoreceptors and sense distinct chemosensory cues.

**Results:**

We describe here that ASEL and ASER also differ substantially in size (soma volume, axonal and dendritic diameter), a feature that is predicted to change the voltage conduction properties of the two sensory neurons. This difference in size is not dependent on sensory input or neuronal activity but developmentally programmed by a pathway of gene regulatory factors that also control left/right asymmetric chemoreceptor expression of the two ASE neurons. This regulatory pathway funnels via the DIE-1 Zn finger transcription factor into the left/right asymmetric distribution of nucleoli that contain the rRNA regulator Fibrillarin/FIB-1, a RNA methyltransferase implicated in the non-hereditary immune disease scleroderma, which we find to be essential to establish the size differences between ASEL and ASER.

**Conclusions:**

Taken together, our findings reveal a remarkable conservation of the linkage of functional lateralization with size differences across phylogeny and provide the first insights into the developmentally programmed regulatory mechanisms that control neuron size lateralities.

## Background

One of the most fundamental aspects of biological control is the regulation of size, on the level of the individual cell, an organ, and the whole organism. Studies in yeast have yielded scores of genes controlling size, many associated with ribosomal protein synthesis [1]. In metazoan organisms, growth and size control are usually studied on the level of either whole organs or even whole organisms, and several genetic mechanisms involved in organism and organ size control have been elucidated [1,2]. For example, signaling pathways triggered by insulin and TGFβ are known to control organismal size [1-4]. Moreover, intriguing links between size control and tumor formation and suppression have been found in the form of genes such as *Myc*, *Brat*, and *TFG *[1,2,5,6].

In spite of these advances, size regulation in the nervous system is poorly understood, even though the size differences of neurons are particularly astonishing. Cross-sectional cell soma size of neurons ranges widely from 0.005 mm to 0.1 mm in mammals. Size in terms of length of axon and dendrites can also hugely differ from neuron type to neuron type, from several microns to several meters within one given mammalian species. Two different nematode species, *Caenorhabditis elegans *and *Ascaris suum *have the same number and types of neurons (their axonal projection patterns are identical as well), yet they differ in soma size and neuronal processes length by several orders of magnitude [7]. Even though the astounding range of neuron sizes in the nervous system has been known for a long time, few genes have been found that specifically control neuronal soma size. One striking case is the gene encoding the phosphatase PTEN, which, when knocked-out, results in a significant increase in neuron soma size, an effect mediated by the kinase mammalian target of rapamycin (mTOR) [8-10]. The importance of the PTEN-mediated neuron-size regulation is illustrated by Lhermitte-Duclos disease, which is characterized by overgrowth of neuronal soma [8,9].

Neuron size regulation is particularly enigmatic when considering size difference between otherwise quite similar neuronal cell types. Such differential size regulation is strikingly apparent in one intriguing and poorly understood context in the nervous system, that of neuronal laterality. In general, nervous systems are morphologically bilaterally symmetric, yet they often are lateralized (left/right asymmetric) in specific functions [11]. That is, groups of neurons located on one side of the brain perform different tasks than their mirror-symmetric neurons on the contralateral side of the brain. This lateralization is evident in many nervous systems across phylogeny, from worms to humans [11-14]. Yet how such asymmetry is genetically programmed is poorly understood. Curiously, in spite of the strong functional lateralization of many brain areas, there are very few genetic correlates to this asymmetry, that is, very few genes are known to be expressed in a left/right asymmetric manner in the adult nervous system of any species [12-14]. However, there is another quite striking correlate to functional asymmetry that has been described in several systems: a difference in soma size of contralateral neuronal ensembles. For example, within several subfields of the human hippocampus, there are regional differences in soma size in the left versus right hemisphere [15]. Intriguingly, these hemispheric soma size differences are abrogated in schizophrenic patients [15]. Left/right asymmetric soma size differences have also been observed within auditory and language-associated regions of the temporal lobe [16]. Similarly, the optic tectum of birds, which is strongly functionally lateralized, displays soma size differences in contralateral neuron types [17,18]. It is, however, not clear how widespread the coupling of functional lateralization and size regulation is. Also, virtually nothing is known about the underlying molecular pathways that control cell size in these left/right asymmetric, neuronal contexts.

**Figure 1:**
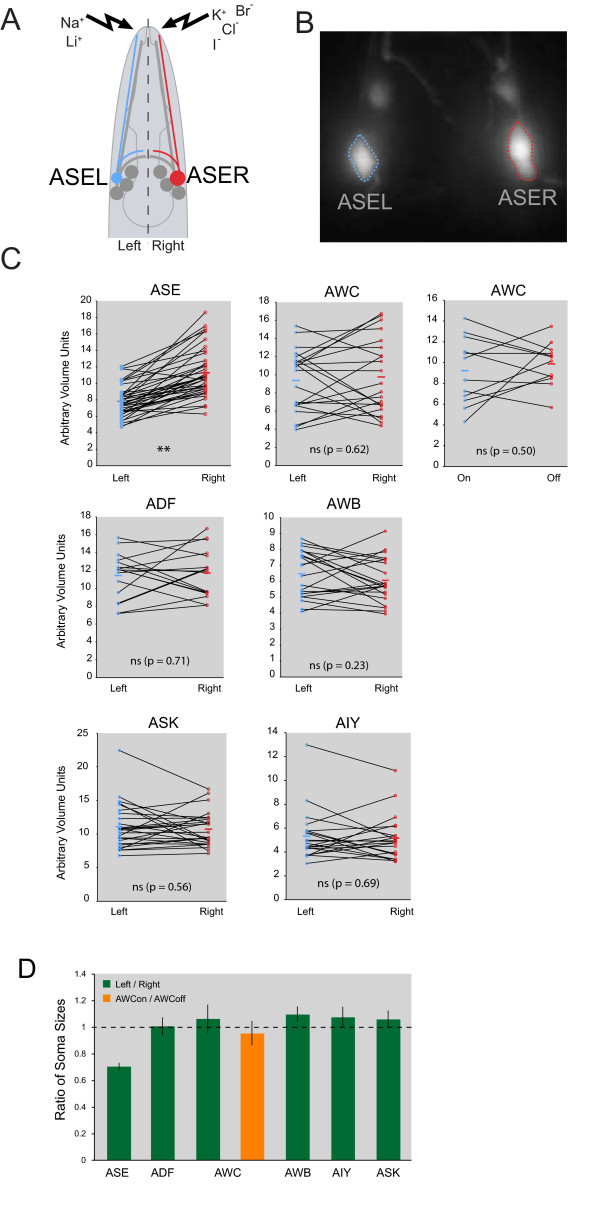
**Examination of lateralized neuron soma sizes in the head ganglia of *C. elegans***. **(A) **Schematic representation of the *C. elegans *head, showing the general symmetric morphology of ASE neurons overlaid with asymmetric function. **(B) **Example of the expression pattern of bilaterally expressed *che-1^prom^*::*gfp*. **(C) **Individual measurements of soma size for several head neuron pairs. Measurements are shown as two open circles; lines connect each individual. Averages of each cell type are indicated as horizontal bars. The AWC neurons, in addition to being measured as a left/right pair, were measured as an AWC^on^/AWC^off ^pair in separate animals, using *str-2::gfp *(*kyIs140) *for AWC^on^/AWC^off ^identification. See Materials and methods for all reporters used. **P < 0.01; ns, not significant. **(D) **Averages of left/right (or AWC^on^/AWC^off^) ratios, generated from the data shown in **(C)**. The dashed line is at a ratio of 1, which indicates the left and right cells are of equivalent volume. Error bars are standard error of the mean.

The nematode *C. elegans *contains an exquisitely well-characterized, largely bilateral nervous system that also displays functional lateralization [12,13] and therefore serves as a good model to investigate the problem of neuronal left/right asymmetry. We investigate here a pair of chemosensory neurons, the ASE neurons (Figure 1A). These two neurons, a left and a right one (ASEL and ASER) are symmetrically positioned in one of the main head ganglia of *C. elegans *and are bilaterally symmetric in many morphological (dendritic morphology, synaptic connectivity) and molecular (gene expression) regards [12,19,20]. However, each neuron senses a distinct spectrum of chemosensory cues and expresses a distinct spectrum of putative chemoreceptors (Figure 1A) [12,21]. Moreover, one neuron (ASEL) responds to upshifts in the concentration of a chemosensory cue, inducing runs in the locomotory behavior of the animal, while the other neuron (ASER) responds to downshifts, inducing reversals of the animal [22]. This lateralization is controlled through a complex bistable system composed of several gene regulatory factors, including regulatory RNAs and transcription factors [23].

Even though its neuronal anatomy has been described in detail, neuronal size has, somewhat curiously, not been studied at any great depth in *C. elegans*. Moreover, it has not been addressed whether functionally lateralized neuron pairs display soma size differences. If this were indeed the case, it may be possible to link genetic mechanisms that control functional lateralization to lateralized size control. We investigate this issue in this paper.

## Results

### The pair of ASE neurons displays size asymmetries

We visualized the ASEL and ASER gustatory neurons in live animals using chromosomally integrated *gfp *reporter gene constructs in which ASE-expressed *cis*-regulatory sequences drive non-localized green fluorescent protein (GFP), which diffuses throughout the entire cell and its processes (Figure 1A). Using two different transgenes (*otIs242 *= *che-1 ^prom^::gfp *and *otIs125 *= *flp-6 ^prom^*::*gfp*), we find that the two neuron soma show consistent and highly stereotyped size differences in adult animals (see Materials and methods for details on size measurements). The volume of the soma of ASER is more than 30% larger than the soma of ASEL (Figure 1).

**Figure 2:**
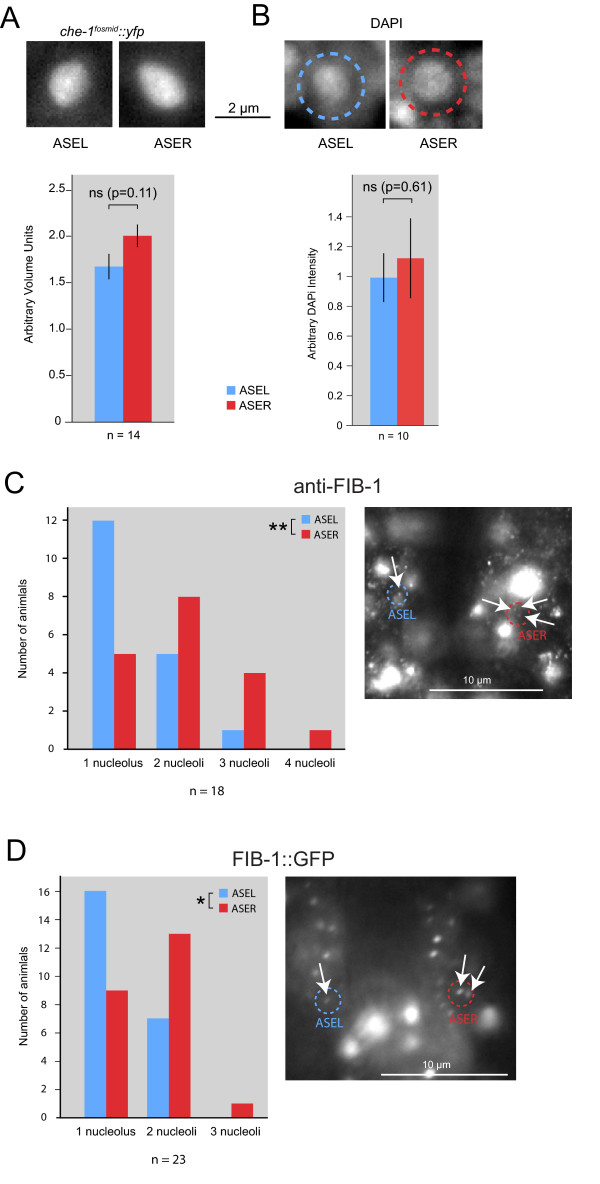
**ASEL/ASER nucleoli number, but not nucleus size or DNA content, is lateralized**. **(A) **Measurement of nuclei sizes in ASEL and ASER using a nuclear-tagged *che-1^fosmid^::yfp *(*otIs188*). Error bars are standard error of the mean (s.e.m.); ns, not significant. A representative pair of nuclei images from one worm is shown. **(B) **Ratio of ploidy in ASEL and ASER. Ploidy was measured by relative DAPI intensity in worms containing a *che-1^prom^::mChOpti *transgene that labels ASE neurons in red. Error bars are s.e.m.; ns, not significant. A representative pair of DAPI images from one worm is shown. **(C) **Measurement of number of nucleoli per cell in ASEL and ASER using an antibody targeting FIB-1. ASE neurons were identified with a *che-1^prom^:: mChOpti *transgene that labels ASE neurons in red. An example image of a worm head is shown; positions of ASEL and ASER are indicated with dashed circles, and arrows point to FIB-1 nucleoli foci. **P < 0.02, as determined by a Wilcoxon signed-rank test. **(D) **Measurement of number of nucleoli per cell in ASEL and ASER using a translational FIB-1::GFP reporter [52]. ASE neurons were identified with a *che-1^prom^:: mChOpti *transgene that labels ASE neurons in red. An example image of a worm head is shown, as in (B). *P < 0.05, as determined by a Wilcoxon signed-rank test.

We next examined the size of specific structures in the soma. Using a *gfp *reporter that is targeted to the nucleus of ASEL and ASER, we find that the volume of the nucleus of ASER is not significantly different from that of the ASEL neuron (Figure 2A). We estimated DNA content (that is, ploidy) of the ASEL versus ASER cell using the standard DAPI stain and observed no significant difference either (Figure 2B). We then visualized the number and size of nucleoli. We find that the ASER neuron contains, on average, more nucleoli (Figure 2C,D).

**Figure 3:**
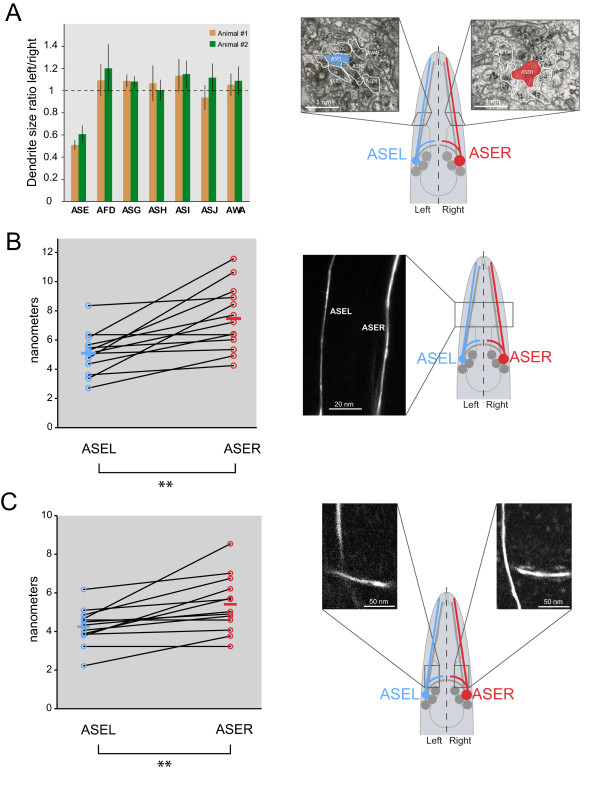
**Lateralized ASE soma sizes are paralleled by lateralized dendrite diameters**. **(A) **Left/right ratios of dendrite sizes derived from electron micrographs. The dashed line is at a ratio of 1, which indicates the left and right dendrites are of equivalent size. Error bars are standard error of the mean for variation within a given dendrite. A representative electron microscopy image of the amphid groups is shown, with the measured neurons outlined and ASEL and ASER highlighted. The scale bar was estimated given the known width of the whole worm at the measured area. n = 7 slices for each individual. **(B) **ASEL and ASER dendrite diameters derived from confocal images (at 63 × magnification) of *otIs242 *(*che-1^prom^*::*gfp*) individuals. Measurements are shown as two open circles; lines connect each individual with each cell type. Averages of left and right are indicated as horizontal bars; **P < 0.01. A representative image of each dendrite pair is shown. **(C) **ASEL and ASER ring neurite diameters derived from confocal images as in **(B)**; measurements for amphid and ring neurites come from the same animals. **P < 0.01. A representative image of each of the dendrite pairs is shown.

Using a set of available electron microscopical sections of the head regions of two different worms, we found that these size differences are not restricted to soma volume, but extend to the relative cross-sectional areas of these neurons. They show an almost twofold difference in cross-sectional area, which translates into a two-fold difference in the volume per unit length (Figure 3A). These results were confirmed with confocal imaging of dendritic diameter using *gfp *reporters (Figure 3B). The axonal projections of ASEL/R into the nerve ring also show lateralities in diameter (Figure 3C). The overall length of the axonal projections and dendrites are the same on the left and right [19].

We also examined a panel of additional neuron pairs in the head ganglia. We examined four additional sensory neuron pairs (AWCL/R, ADFL/R, AWBL/R, ASKL/R) and one interneuron pair (AIYL/R; the main postsynaptic target of ASEL/R). We found that even though there was some variation in individual animals, none of these neurons showed, on average, any indication of a consistent laterality in soma size (Figure 1C,D). This notion was corroborated by an analysis of sensory dendrite diameter, in which we also found no significant sidedness (Figure 3A), again in contrast to the situation with ASEL/R.

We examined the AWCL/R case in more detail. Like the ASEL/R gustatory neuron pair, this olfactory neuron pair is known to be functionally lateralized. The left versus right neurons sense different sensory cues and process information differentially [13,24,25]. However, in contrast to ASEL/R laterality, which is deterministic (that is, 100% invariant; a phenomenon called 'directional asymmetry') [26], AWCL/R asymmetry is stochastic (a phenomenon called 'antisymmetry') [26]. This lateralization can be visualized with two distinct putative odorant receptors, *str-2 *and *srsx-3 *[27]. In 50% of animals *str-2 *is expressed in the AWCL, while in the other 50% it is expressed in AWCR. *srsx-3 *shows the complementary pattern. The *str-2*-expressing cell has traditionally been called the AWC^on ^cell [24]. Even though, on average, AWC soma showed no laterality, we tested whether the AWC^on ^or AWC^off ^cell may correlate with a specific relative size. However, this is not the case (Figure 1C,D).

Taken together, the functionally lateralized ASEL/R neuron pair shows a consistent soma size laterality that is paralleled by axonal, dendritic, and nucleolar lateralities, but not by lateralities in nuclear size or DNA content. The neuron pairs that we examined for lateralities included neuron pairs in physical proximity to ASEL/R and/or related by common ancestry (that is, lineage). A lack of directional asymmetry in these related neuron pairs illustrates that it is not simply the case that one side ('hemisphere') of the worm is larger than the other, but rather that neuron size is regulated in a neuron-type-specific manner. We also note that absolute size measurements of other neuron pairs differ from neuron type to neuron type, with the larger ASER not being larger than other neuron pairs and the smaller ASEL not being smaller than yet other neuron pairs. It is therefore not obvious as to whether the size difference between ASEL and ASER is due to 'overgrowth' of ASER or 'growth inhibition' of ASEL.

### Size differences translate into distinct electrophysiological properties

**Figure 4:**
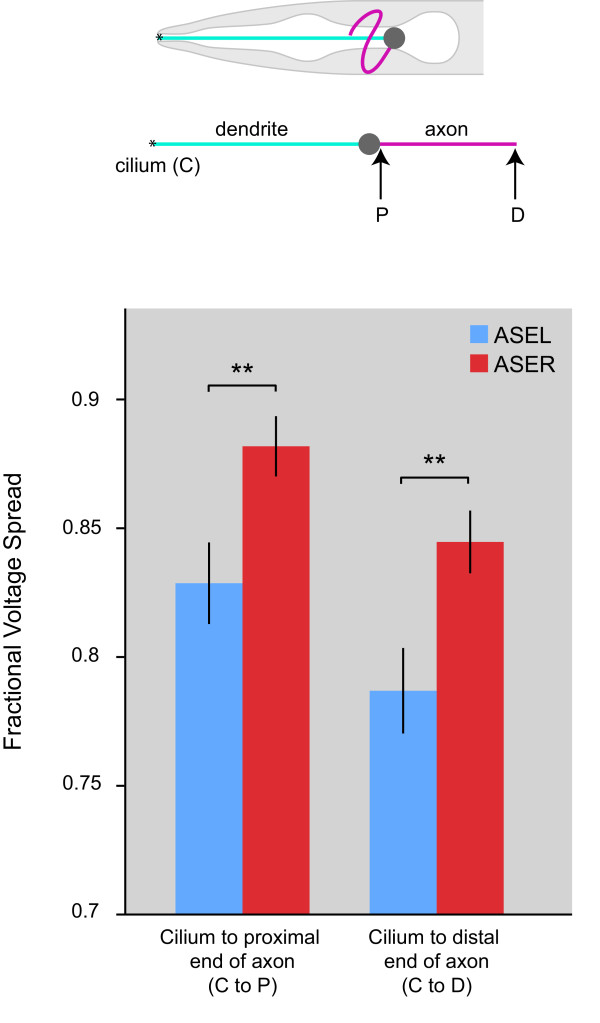
**Size lateralization translates into distinct electrophysiological properties of ASEL versus ASER**. Voltage at the proximal and distal ends of the axon (indicated in the schematic on top) is shown in response to a unit depolarization of the distal tip of the dendrite, representing the sensory cilium ('C'). Voltages were computed using standard cable theory. On the y-axis, a value of 1 indicates the voltage that would be expected in an isopotential neuron. Error bars are s.e.m.; **P < 0.01.

One of the most likely functional consequences of a difference in size is a difference in the passive spread of voltage from one end of a neuron to the other. To assess whether the observed left-right differences in neurite diameters are theoretically sufficient to produce a significant difference in voltage spread, we modeled ASE neurons as a pair of cylindrical cables representing the dendrite and axon. The cables were joined at one end and sealed at the other. The soma was omitted because it is too small to affect the extent of voltage spread [28]. Voltage spread is a function of the ratio *R *of membrane and axial resistivity as well as the anatomical dimensions. *R *was set to the value obtained in a previous analysis of ASER neurons [28]. Here we assume that the effective passive electrical properties of ASEL and ASER, including the value of *R*, are the same for small depolarizations in the likely operating range of the neurons. Partial support for this assumption is provided by the fact that the steady state current-voltage relationships of these neurons are nearly identical in their operating range. Dendrite and axon lengths were measured in confocal reconstructions from GFP-labeled ASE neurons in unfixed animals (dendrite, 116 μm, n = 28; axon, 80 μm, n = 18). The diameter of the dendrites and axons of ASEL and ASER neurons were measured separately in each of 13 worms (Figure 3B,C). For each worm, we used standard cable theory [28,29] to compute the steady-state voltage at the beginning or end of the axon in response to a unit depolarization of the distal tip of the dendrite (representing the sensory cilium where sensory transduction is believed to occur in real ASE neurons). We found small but significant differences in the extent of voltage spread at both locations (Figure 4). As output synapses from ASEL and ASER neurons reside along the entire length of their axons, we conclude that differences in process diameters could result in stronger outputs from ASER neurons.

### Size laterality does not depend on sensory activity, but is embryonically programmed by the *che-1 *transcription factor

**Figure 5:**
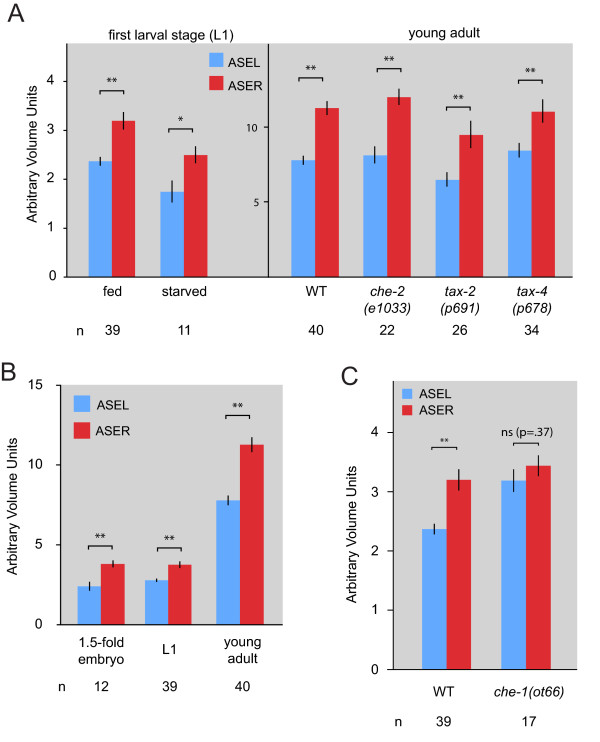
**Size lateralization is not activity dependent but developmentally programmed**. **(A) **Measurements of ASEL and ASER soma volumes in worms with less neuronal activity. Neuronal activity was reduced either by hatching embryos into ddH2O and leaving them for approximately 16 hours (labeled starved) or by mutant analysis. Starved worms had to be measured as L1s, as the worms will not grow past the L1 stage in the absence of food. All others were measured as adults. Wild-type (WT) volumes are shown for comparison. Error bars are standard error of the mean (s.e.m.); *P < 0.05, **P < 0.01. **(B) **Measurements of ASEL and ASER volume sizes during development. Error bars are s.e.m.; **P < 0.01. **(C) **Measurement of ASEL and ASER volume sizes in a *che-1 *mutant. Error bars are s.e.m.; **P < 0.01. ns, not significant.

The soma size lateralities in the optic tectum of birds correlate with loci of functional lateralities, and those functional lateralities are dependent on visual input, that is, neuronal activity [11,17,18]. We therefore tested whether activity of the ASE neurons has an impact on their size differences. We examined soma size lateralities in a number of mutants in which the ASE neurons are not able to sense or transduce sensory stimuli. We observed no effect on soma size laterality (Figure 5A). Keeping animals in a sensory-deprived environment by hatching them in water also does not affect soma size lateralities (Figure 5A). These findings suggest that rather than being activity-dependent, size lateralities may be developmentally programmed. To test this notion, we examined ASEL/R size laterality not just in the adult, but also at earlier stages. We indeed find that already at the first larval stage, right after hatching, the differences in size between the two neurons is already as apparent as in the adult (Figure 5B). Going back to the 450-minute stage of embryogenesis-100 minutes after the ASE neurons are formed-we already observe size differences. The observation of differential size regulation occurring in the *C. elegans *embryo is somewhat unexpected as, in contrast to the enormous size increase of all cell types after hatching, there is in general little overall cell growth in embryos. Rather, as the overall volume of the embryo is constant, every cell division results in smaller daughter cell sizes.

To begin analyzing the genetic mechanisms that underlie these size differences, we first used a genetic background in which the ASEL/R neurons fail to be appropriately specified. The ASEL/R-specific *che-1 *Zn finger transcription factor is required for the correct development of ASEL/R neurons; in *che-1 *mutants, ASEL/R neurons are not functional (that is, animals are not able to chemotax to water-soluble attractants, hence the name *che*), and fail to express scores of genes that are normally expressed in ASE, yet the ASE neurons are still generated [20,30,31]. Measuring the size of ASE neurons in *che-1 *mutants, we find that the soma differences of ASEL and ASER are eliminated (Figure 5C). Left/right size differences are therefore programmed through the activity of the *che-1 *transcription factor.

### Gene regulatory factors that control functional laterality also control size asymmetry

**Figure 6:**
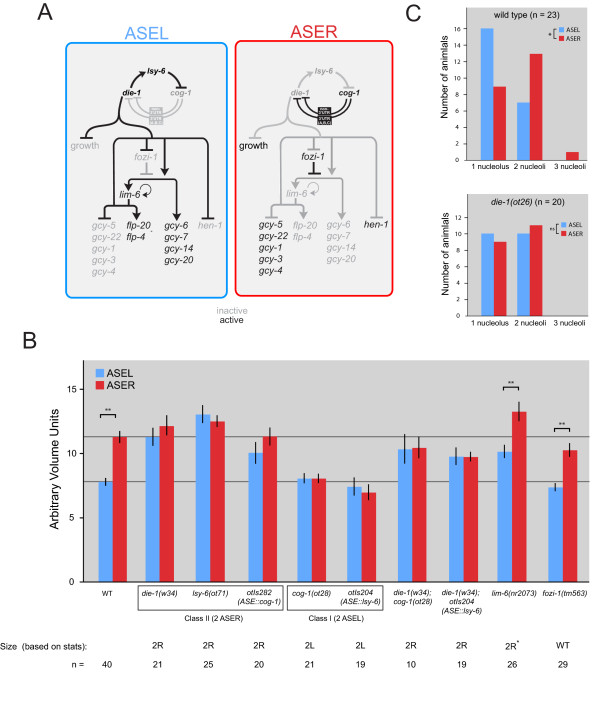
**Size control is downstream of *die-1 *but independent of *lim-6 *and *fozi-1***. **(A) **Gene regulatory network controlling ASE asymmetry [53]. **(B) **Measurements of ASEL and ASER volumes in various mutant backgrounds. Error bars are standard error of the mean. Based on statistical comparison, the measured sizes correspond to the following: 'WT' = the mutant ASEL volume is not significantly different from a wild-type ASEL and the mutant ASER volume is not significantly different from a wild-type ASER; '2L' = both cells are significantly different from ASER and not significantly different from ASEL; '2R' = both cells are significantly different from ASEL and not significantly different from ASER; '2R*' = both cells are significantly different from ASEL and not significantly different from ASER, but are significantly different from each other; all comparisons to wild-type cells utilize the Bonferroni correction. **P < 0.01. **(C) **The effect of loss of *die-1 *on nucleoli number in ASEL versus ASER. Wild-type control is repeated from Figure 2 and shown for comparison only. *P < 0.05, as determined by a Wilcoxon signed-rank test; ns, not significant.

We next turned to a set of genes that we have previously identified as controlling the functional left/right asymmetry of the ASE neurons [23]. A complex regulatory system, composed of transcription factors and regulatory RNAs, controls the left/right asymmetric expression of distinct putative chemoreceptors of the *gcy *gene family in ASEL versus ASER (Figure 6A). The activity of what we termed 'class I' regulatory genes promotes ASER fate, and their loss leads to a conversion of ASER to ASEL. 'Class II' regulatory genes have the opposite activity; they promote ASEL fate and their loss leads to a conversion of ASEL to ASER. Class I and class II genes cross-inhibit each other's activities (Figure 6A).

We first analyzed ASE soma size lateralities in three different genetic contexts in which both neurons are transformed to the ASER fate ('2 ASER'; as assessed by *gcy *chemoreceptor gene expression). We used animals carrying loss-of-function mutations in the ASEL inducers *die-1 *(a Zn finger transcription factor) and *lsy-6 *(a miRNA), and transgenic animals in which the ASER-inducer *cog-1 *(a homeobox gene) is ectopically expressed in both ASE neurons. We find that in all three genetic backgrounds, both ASE neurons now adopt the larger size that is normally characteristic of ASER (Figure 6B). Similarly, we analyzed ASE soma size lateralities in two different genetic contexts in which both neurons are transformed to the ASEL fate ('2 ASEL'; as assessed by *gcy *chemoreceptor gene expression), namely in animals carrying loss of function mutation in the ASER inducers *cog-1 *and in transgenic animals that ectopically express the ASEL-inducer *lsy-6 *bilaterally in both ASE neurons. In both genetic backgrounds, both ASE neurons now adopt the smaller size that is normally characteristic of ASEL (Figure 6B). The effect of *die-1 *manifests itself not only on the soma size difference of ASEL/R, but also on difference in the number of nucleoli; they become bilaterally symmetric in the *die-1 *mutant (Figure 6C).

ASEL and ASER inducers act in a feedback loop [32]. We sought to determine which genes provide the output from this loop to size control. For the determination of left/right asymmetric chemoreceptor expression, *die-1 *is the output, as the effect of *die-1 *on all previously known lateralities is epistatic to any genetic manipulations in the loop [32]. We performed similar epistasis experiment, scoring asymmetric soma size. We find that *die-1 *is epistatic to both manipulations of *cog-1 *and *lsy-6 *activity (Figure 6B). That is, the '2 ASEL size' phenotype of either *cog-1(-) *or *lsy-6 *misexpression is reverted to the '2 ASER size' phenotype in a *die-1(-) *background.

The two transcription factors *lim-6 *(a LIM homeobox gene) and *fozi-1 *(a Zn finger transcription factor) act downstream of *die-1 *as effector genes, regulating a subset of left/right asymmetric features of ASEL and ASER (Figure 6A) [32,33]. We find that these regulators have no impact on the ASEL/R soma size differential (Figure 6B).

Taken together, these findings show that size control is tightly controlled by a genetic regulatory mechanism that defines other aspects of laterality of the ASEL and ASER neurons as well. The control of left/right asymmetric size and chemoreceptor expression does, however, branch out downstream of *die-1 *(Figure 6A), as *lim-6 *and *fozi-1 *affect chemoreceptor expression but not size. We hypothesize that *die-1 *regulates either directly or indirectly the expression of effector genes that control size.

### A candidate gene approach identifies the nucleolar protein FIB-1 as a size regulator

**Figure 7:**
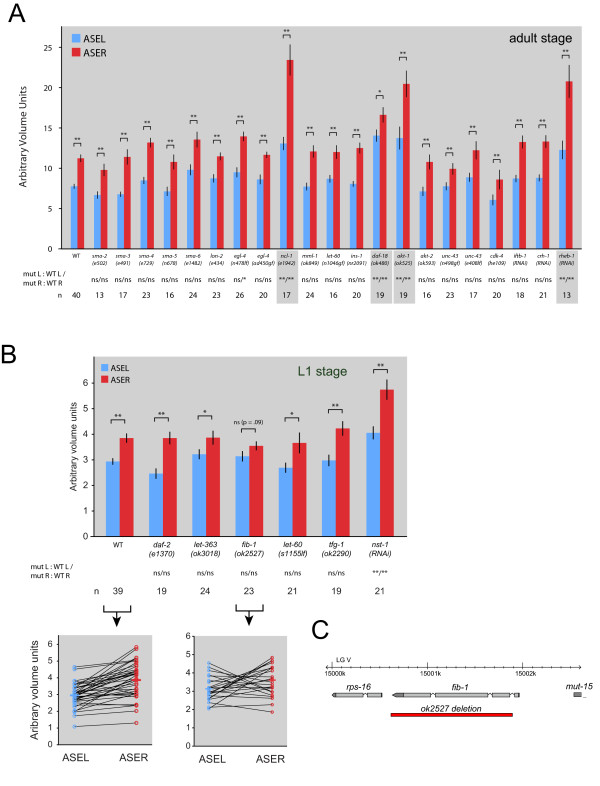
**The impact of size control genes on ASEL/R size**. **(A) **ASEL/R soma size differences in various genetic backgrounds. See Table 1 for more details on identity of genes and for references on individual genes. Error bars are standard error of the mean (s.e.m.); *P < 0.05, **P < 0.01 for comparisons within a genotype. Statistical comparisons are also shown between mutant and wild-type (WT) ASEL and mutant and wild-type ASER; these use the Bonferroni correction. Mutants that affect both ASEL and ASER size are highlighted in grey. **(B) **Measurements as in (A), but for those mutants that must be measured at the first larval state due to growth arrest or death shortly after that state. Error bars are s.e.m.; *P < 0.05, **P < 0.01 for comparisons within a genotype. Statistical comparisons are also shown between mutant and wild-type ASEL and mutant and wild-type ASER; these use the Bonferroni correction. Wild-type (N2) and *fib-1 *mutant data are also shown on a single animal basis, with ASEL and ASER represented in two open circles, as shown in Figure 1. **(C) **Structure of the *fib-1 *deletion allele.

The impact of the DIE-1 and CHE-1 transcription factors on lateralization of soma size is presumably mediated by gene(s) that are under control of these factors and possibly expressed in a left/right asymmetric manner. In an attempt to identify these effector genes, we tested a large number of candidate genes for an effect on ASEL/R soma size differences. These candidates encode proteins that have, in various different systems, been implicated in controlling cell size. The candidate genes that we tested-a total of 24 loci (some tested both with gain-and loss-of-function alleles)-are listed in Table 1 and results are shown Figure 7. Among the tested strains are animals mutant components of the insulin receptor-like signaling system, the *C. elegans *Myc homolog *mml-1 *[34], regulators of ribosomal RNA synthesis like Brat/*ncl-1 *[1], *sma *and *lon *genes [4], the *C. elegans *homolog of the nucleolar protein Fibrillarin, FIB-1, and a recently discovered set of genes involved in body size control in worms (CREB-like gene *crh-1*, nucleostemin/*nst-1*, translational initiation factor eIF2B/*iftb-1*, tumor suppressor gene *TFG/tfg-1*) [6]. We also tested the impact of a calcium-dependent pathway that in other systems is involved in cell swelling in response to external/environmental challenges ('regulatory volume decrease') [35].

**Table 1:** Background information on candidate genes tested for ASEL/R size differences

Reason for testing	Genes tested	Identity
Controls overall body and/or cell size in *C. elegans *[4,6,54,55]	*sma-2*	Smad (TGFR signaling)
	*sma-3*	Smad
	*sma-4*	Smad
	*sma-5*	MAPK
	*sma-6*	Kinase receptor
	*lon-2*	Glypican
	*egl-4*	cGMP-dependent kinase
	*ncl-1*	RBP (Brat tumor suppressor)
		
Controls cell size in *C. elegans *and other systems [6]	*nst-1*	Nucleostemin
	*iftb-1*	eIF2B
	*tfg-1*	TFG oncogene
	*crh-1*	CREB/ATF-family
		
Controls cell size in other systems [1,34,56-58]	*mml-1*	Myc
	*let-60*	Ras
	*daf-2*	Insulin/IGF-receptor
	*ins-1*	Insulin ligand**^a^**
	*daf-18*	PTEN phosphatase
	*let-363*	TOR kinase
	*akt-1*	Protein kinase B
	*akt-2*	Protein kinase B
	*fib-1*	Fibrillarin
	*rheb-1*	GTPase
	*cdk-4*	Cyclin-dependent kinase
		
Regulatory volume control in other systems	*unc-43*	CaMKII

**^a^***ins-1 *is only one of several insulin ligands in the worm; we specifically tested this one as it is known to be released from a postsynaptic ASE target to affect InR signaling in ASER in the context of learning and memory [59]. InR: Insulin/IGF-receptor.

We found that reduction or elimination of only some of the candidate size regulators affect overall ASEL and ASER size (Figure 7A,B). These include the phosphatase PTEN, the kinase AKT, the Brat tumor suppressor Brat/Ncl-1 and the small GTPase Rheb-1, but surprisingly, not canonical size regulators, such as the insulin/IGF-1 receptor (Figure 7A,B). Of all the mutant animals tested, only one eliminated the difference in soma size between ASEL and ASER (Figure 7B). These animals carry a deletion allele, *ok2527 *(kindly provided by the Oklahoma *C. elegans *knockout consortium; Figure 7C) that eliminates the nucleolar protein Fibrillarin/FIB-1, an RNA methyltransferase involved in ribosome biogenesis [36]. This finding is in accordance with the observation that ASER contains more FIB-1 positive nucleoli than ASEL (Figure 2). Linking FIB-1 accumulation to the upstream gene regulatory factors, we find that in *die-1 *mutants, the number of FIB-1(+) nucleoli increases in ASEL (Figure 6C).

Even though *fib-1 *is required for the manifestation of the size differences, it is not sufficient, as we did not observe any effect on the size differential in transgenic animals that overexpress *fib-1 *bilaterally in both ASEL and ASER using the *ceh-36 *promoter (four transgenic lines tested; data not shown). We also note that loss of *fib-1 *has no effect on left/right asymmetric chemoreceptor expression (*gcy-5 *and *gcy-7; *data not shown), corroborating the notion that size control can be decoupled from other aspects of ASEL/R laterality. In conclusion, our candidate gene analysis has uncovered a protein with a function in nucleolar biogenesis required for left/right differential size laterality in the nervous system.

## Discussion

We describe here a developmentally programmed size laterality of a functionally lateralized neuron pair. It is striking that the theme of lateralized soma sizes in functionally lateralized brain regions is conserved from higher vertebrates-for example, the optic tectum in chick [17,18]-to a simple invertebrate like *C. elegans*.

The theoretical differences in passive voltage spread presented here (Figure 4) could have significant functional consequences. Other things being equal, one would expect stronger synaptic outputs from ASER in response to the same level of depolarization in the cilia of two neurons. Notably, it can be shown from first principles that for chemotaxis in a radial gradient, "off cells" like ASER (i.e. neurons responding to a decrease of a signal) are sufficient, whereas "on cells" like ASEL (i.e. neurons responding to an increase of a signal) are not [37]. Thus, worms with stronger ASER outputs would enjoy a selective advantage, which may have resulted in an increase in ASER size. If validated experimentally, differential voltage spread would join a growing list of several distinct properties of the ASEL versus ASER neurons, including differential sensation of taste cues, differential chemoreceptor expression, differential response to upsteps (ASEL) versus downsteps (ASER) of chemosensory cues and differential contributions to spatial orientation behaviors [36,38]. These features are layered upon otherwise largely symmetric characteristics of ASE [20]. However, in contrast to the invariant left/right asymmetric expression of chemoreceptors, we note that the ASER > ASEL size differences are only observed when averaged over a population. That is, there are individuals in which either no differences in size are observed or in which the size asymmetry is reversed. Whether this is due to experimental error or is an indication of distinct chemosensory capacities of individual animals within a population remains to be determined.

**Figure 8:**
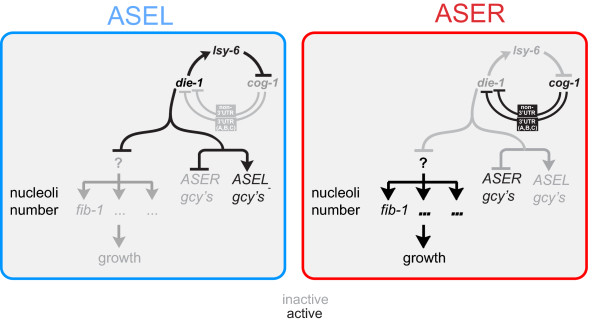
**Model for size regulation in ASEL/R**. *die-1 *gene activity controls left/right asymmetric functional features, such as asymmetric expression of the putative *gcy *chemoreceptors, and is required for the asymmetric size of ASEL versus ASER as well as the asymmetric number of FIB-1(+) nucleoli in ASEL versus ASER. The effect of *die-1 *on size cannot solely be explained through regulation of *fib-1*, as *fib-1 *is required but not sufficient to impose ASER size. *die-1 *may therefore regulate the expression of other genes involved in size control. *die-1 *may regulate *fib-1 *expression directly or indirectly (as indicated with the question mark).

We provide here three mechanistic insights into how differential size regulation is achieved. First, we find that size asymmetries are not activity-dependent, but developmentally controlled. Second, we have identified a transcriptional regulator, the Zn finger transcription factor DIE-1 (as well as its upstream regulators), which controls size laterality. The involvement of *die-1 *in controlling size parallels its involvement in controlling lateralized chemoreceptor expression. However, transcription factors acting downstream of *die-1*, namely the *lim-6 *LIM homeobox gene and the *fozi-1 *Zn finger factor, which also affect chemoreceptor expression, do not affect differential size regulation. Regulatory pathways controlling size and chemoreceptor expression therefore branch downstream of *die-1 *(summarized in Figure 8). Third, we have identified the functionally as yet uncharacterized *C. elegans *fibrillarin gene *fib-1 *as a gene required for ASEL/R size laterality. *fib-1 *encodes a phylogenetically conserved RNA methyltransferase involved in ribosome biogenesis whose human homolog is a nucleolar autoantigen for the non-hereditary immune disease scleroderma [39]. Our demonstration that loss of *fib-1 *results in alterations on cell size may not be unexpected, given that yeast fibrillarin has been found to control pre-rRNA processing, pre-rRNA methylation and ribosome assembly [40] and that nucleolar size and ribosomal biogenesis have been previously linked to cell size control [1], but our results nevertheless provide the first direct implication of fibrillarin in cell size control and they also place fibrillarin activity and nucleolar size into a previously unknown cellular and functional context.

*fib-1 *acts downstream, and is therefore a target of the *die-1 *Zn finger transcription factor, a conclusion based on our observation that the number of FIB-1(+) nucleoli increases - together with overall size - if normal *die-1 *expression in ASEL is lost. At this point, we can not tell whether the *fib-1 *locus is a direct transcriptional target of DIE-1 or whether differential FIB-1 accumulation in ASEL versus ASER is an indirect consequence of DIE-1 function in ASEL (or absence thereof in ASER). *fib-1 *is unlikely to be the sole (direct or indirect) target of DIE-1 in the context of size control since *fib-1*, unlike *die-1*, is not sufficient to impose ASER size. Work in yeast and flies has amply demonstrated that the genes encoding nucleolar proteins involved in ribosome biogenesis, such as fibrillarin, are co-regulated through common transcriptional control mechanisms ('Ribi regulon') [41-44]. Several distinct types of transcription factors are involved in controlling the Ribi regulon, such as the yeast Forkhead like protein Fhl1 or, in metazoans, the Myc transcription factor [42-44]. DIE-1 may either be directly involved in such a co-regulatory mechanism or may be involved in indirectly triggering such a mechanism via intermediary regulators (Figure 8). DIE-1 therefore joins the ever-growing list of transcriptional regulators of cell size; however, the role of DIE-1 in size regulation may be highly context dependent, as *die-1 *mutants do not display any gross defects in animal size.

Our analysis of candidate size regulators has also identified a series of genes that control overall neuron size in a bilaterally symmetric manner (that is, both ASEL and ASER are affected). Given the paucity of known size regulators in the nervous system, some of our partially unexpected results raise questions and provide a starting point for future analysis. As expected from work in other systems [8-10], *daf-18*/PTEN mutants show increased neuron size. However, a null mutation in the insulin/IGF-like receptor in worms, *daf-2*, does not affect neuron size, even though the same signaling system does have profound effects on size and growth in other organisms [45]. Yet, loss of another gene in the *daf-2 *pathway, the Ser/Thr kinase *akt-1 *does significantly affect the size of both ASEL and ASER, suggesting that AKT may be coupled to a distinct upstream input. However, unlike in other systems, in which AKT negatively regulates size [46], ASEL and ASER size is increased in *akt-1 *mutants. A similar, unexpected 'sign reversal' is observed in animals lacking the size regulators *rheb-1*, a small GTPase, or the nucleolar protein nucleostemin/*nst-1*, both known to be required to promote growth in other systems [47,48], but apparently inhibiting growth of both ASE neurons. Other known size regulators, such as *cdk-4 *[49], do not effect ASEL/R neuron size at all. We also found no effect of removing the canonical size regulator *let-363/*TOR; however, these animals could only be scored at the first larval stage due to later larval lethality. The maternal load of TOR may have rescued any potential size regulatory effect. The same caveat holds for interpretation of the lack of effect of removing *let-60/*Ras and *tfg-1*/TFG. Lastly, we note that a transforming growth factor-β signaling pathway previously reported to control overall animal size in *C. elegans *[4] does not affect ASE neuron size, demonstrating that overall animal size is decoupled from neuron size.

In conclusion, we have provided some of the first mechanistic insights into how lateralized neuron size is controlled and we have set a theoretic framework for the type of impact such size difference may have on neuron function. It is conceivable that lateralized neuron size differences in vertebrates may also be controlled via nucleolar mechanisms [50], a notion that is not a matter of course since known cell size control pathways do not necessarily work through regulation of ribosomal and hence nucleolar mechanisms [43]. Our findings also raise the possibility that lateralized neuron size control may be uncoupled from more canonical mechanisms of size control in other cell and tissue types. This is because we find that asymmetric neuron size control is established at a stage (embryo) when no other tissues undergo the generic growth that is characteristic of late embryonic and larval growth and because asymmetric neuron size control does not involve many of the canonical body size regulators. The identification of direct target genes of the *die-1 *transcription factor, the regulator we found to impinge on the ASEL/R size differential, will provide more insights into this pathway in the future.

## Materials and methods

### Transgenic reporter strains

The following transgenes were used to measure neuron soma sizes: ASEL/R, *otIs125 = flp-6 ^prom^::gfp; otIs242 = che-1^prom^::gfp*; AWCL/R, *otIs151 *= *ceh-36::dsRed2*; AWC^on/off^, *otEx9961 *= *srsx-3::TagRFP*; AWCL/R, *oyIs28 = odr-1::gfp*; ADFL/R, *zdIs13 = tph-1::gfp*; AWBL/R, *kyIs104 = str-1::gfp*; ASKL/R, *otEx4302 = sra-9::gfp*; AIYL/R, *otIs173 = ttx-3^prom^::gfp*. ASE nuclear size was measured with *otIs188 *(*che-1^fosmid^::yfp*).

### Measurements of ASE features

For the soma or nuclear size measurement, transgenic worms, harboring neuron-type specifically expressed reporter constructs are picked at the desired stage (either L1 or adult) and examined using an Axioplan 2 microscope and a Sensicam QE camera controlled by Micro-Manager software [51]. Worms were rolled on the cover slip such that ASEL and ASER were in the same plane (dorso-ventral view), and stacks were made with a 63 × oil-immersion objective at 1 μm depth. The stacks were analyzed using ImageJ software, where the contrast of the cell was chosen such that the fluorescence intensity did not impinge on neighboring cells, and the ImageJ plugin Voxel Counter was used to count the number of pixels for each cell. GFP intensity was normalized by cropping stacks around each cell separately and adjusting the brightness levels of the two stacks such that the maximum intensity level of each stack was reset to one standard. Statistical analysis of the relative sizes within a given strain was also performed by using a paired two-tailed *t*-test; significance was determined using the Bonferroni correction. For sets of experiments where n ≥ 3, we employed the Bonferroni correction: instead of using thresholds of *P *< 0.05 or *P *< 0.01, we used stricter *P*-value thresholds of *P *< 1-((1-0.05)^1/n^) and *P *< 1-((1-0.01)^1/n^), respectively, where n is the number of experiments in a given set. We measured cross-sectional diameters in the electron micrographs by tracing each dendrite in ImageJ and using the Measure tool.

We measured ploidy by ethanol fixation followed by DAPI staining either *otIs151 *(*ceh-36^prom^::rfp*) or *otIs232 *(*che-1::mChopti*) for ASE cell identification. Image stacks of DAPI-stained worms were taken using the method described above. We measured DAPI intensity as a proxy for DNA amount and report the data as relative DAPI intensities. We used freeze fracture followed by methanol/acetone fixation for immunostaining.

To determine nucleoli size and number, we used *cguIs001 *(*fib-1::gfp*) [52] and an antibody against Nop1p (FIB-1) from EnCor BioTechnology (#MCA-38F3, Gainesville, FL, USA) at a 1:200 dilution, detected with a 1:200 dilution of an anti-mouse (Invitrogen #A-21202, Carlsbad, CA, USA") secondary antibody.

## Abbreviations

GFP: green fluorescent protein; IGF: insulin-like growth factor.

## Competing interests

The authors declare that they have no competing interests.

## Authors' contributions

AG conducted all experiments shown in this paper, SS conducted some initial size experiments, SL guided the voltage analysis, OH initiated and supervised this study, and AG and OH wrote the paper.
